# 

**Published:** 1845-01

**Authors:** 


### CONTENTS OF No. XXXVII

OF THE

### British and Foreign Medical Review.

JANUARY, 1845.

---

### Part First.—Analytical and Critical Reviews.

PAGE

Art. I.—Illustrations of the Theory and Practice of Ventilation, with Remarks on Warming, Exclusive Lighting, and the Communication of Sound. By D. B. Reid, m.d. . . . . . . . 1

Art. II.—Lectures on the more important Diseases of the Thoracic and Abdominal Viscera, delivered in the University of Pennsylvania. By N. Chapman, m.d. 21

Art. III.—1. Die Psychischen Krankheiten, und die damit verwandten Zustände in Bezug auf die Rechtspflege. Von J. H. Hoffbauer . . 33

On Mental Diseases and their Legal Relations. By J. H. Hoffbauer.

2. A Treatise on the Medical Jurisprudence of Insanity. By J. Ray, m.d., Superintendent of the Maine Insane Hospital . . . ib.

3. Remarks on the Impunity of certain Attempts to Murder. By T. Mayo, m.d. ib.

Art. IV.—1. Das Pest-Contagium in Egypten und seine Quelle, nebst einem Beitrage zum Absperr-systeme. Von J. F. Grohmann, Med. Dr. des Kaiserthums Oesterreich und Königreichs Sachsen, &c. . . 41

Plague-Contagion in Egypt, and its Source, with a Supplement on the Quarantine System. By J. F. R. Grohmann, m.d. of the Empire of Austria and Kingdom of Saxony, &c.

2. Risposta ai sette Quesiti concernenti la Peste bubonica orientale. Del Dot. Francesco Grassi, Cavaliere di piu ordini, gia Medico-Chirurgo in capo dello Spedale generale della marina di guerra Egizia, &c. &c. . . ib.

Answer to the Seven Questions concerning the Oriental Bubonic Plague. By Dr. Francesco Grassi, Knight of several orders, Chief Medical Officer of the General Hospital of the Egyptian Navy, &c.

3. Correspondence relative to the Contagion of Plague, and the Quarantine Regulations of Foreign Countries, 1836-43. Presented to the House of Commons in pursuance of the Addresses of the 28th August 1841, and 15th March 1842 . . . . . . ib.

Art. V.—Traité d'Hygiène publique et privée. By Michel Lévy, Médecin ordinaire de premier class, &c. Tome Premier . . . 51

A Treatise on Hygiene, public and private. By M. Lévy, Physician to the Forces, Professor of Hygiene and Forensic Medicine, &c. &c. Vol. I.

Art. VI.—1. Lehrbuch der Geburtshülfe für die Hebammen im Grossherzogthume Baden. Von Franz Carl Naegele, Grossherzoglich Badischem Geheimerathe, ordentl. offentl. Professor der Medizin und Geburtshulfe, Direktor der Entbingdungs-anstalt zu Heidelberg, &c. . . . 58

Compendium of Midwifery for Midwives in the Grand Duchy of Baden. By F. C. Naegele, m.d., Professor of Medicine and Midwifery, and Director of the Lying-in Hospital at Heidelberg.

§

4296

PAGE

2. Lehrbuch der Geburtshülfe. Von Dr. HERMANN FRANZ NAEGELE. ausserordentlichem Prof. der Medizin an der Universität Heidelberg. Erster Thiel—Physiologie und Diätetik der Geburt . . . . . 58

Compendium of Midwifery. By Dr. H. F. NAEGELE, Professor extraordinary of Medicine at the University of Heidelberg. Part First—Physiology and Treatment of Labour.

ART. VII.—Half-yearly Reports of the Government Charitable Dispensaries established in the Bengal and North-western Provinces, from 1st August 1840, to 31st January 1842 . . . . . 72

ART. VIII.—Ueber die Pacinischen Körperchen an den Nerven des Menschen und der Säugethiere. Von J. HENLE und A. KÖLLIKER . . 78

On the Pacinian Corpuscles on the Nerves of Men and Mammalia. By J. HENLE and A. KÖLLIKER.

ART. IX.—1. De la Puberté et de l'Age Critique chez la Femme, au point de vue physiologique, hygiènique, et médical, et de la Ponte périodique chez la Femme et les Mammifères. Par M. A. RACIBORSKI . . 84

An Essay on the Physiology, Hygiene, and Medical Management of the Epochs of Puberty and Cessation of the Menses in Women; and on the Periodical Discharge of Ova in the Human Female and in Mammifera. By M. A. RACIBORSKI.

2. Beweis der von der Begattung unabhängigen periodischen Reifung und Loslösung der Eier der Säugethiere und des Menschen als der ersten Bedeingung ihrer Fortpflanzung. Von Th. S. W. BISCHOFF . . ib.

Proof that the Periodical Maturation and Discharge of the Ova, independently of Coitus, in Mammalia and the Human Female, constitute the essential condition of their Propagation. By Prof. BISCHOFF.

ART. X.—Abhandlung über Spinal Irritation, &c. Von LUDWIG TÜRCK, Doctor der Medicin, &c. . . . . . . 96

A Treatise on Spinal Irritation; the Result of Observations made principally in the General Hospital of Vienna. By Dr. L. TÜRCK, &c.

ART. XI.—On the Changes induced in the Situation and Structure of the Internal Organs, under varying circumstances of Health and Disease; and on the Nature and External Indications of these Changes. (Transactions of the Provincial Med. and Surg. Association. Vol. XXI.) By FRANCIS SIBSON 103

ART. XII.—Akesios. Blicke in die ethischen Beziehungen der Medicin. Von K. F. H. MARX . . . . . . 121

Akesius; a Glance into the Ethical Relations of Medicine. By K. F. H. MARX.

ART. XIII.—De la Mortalité à Bruxelles, comparée à celle des autres grandes Villes. Par ED. DUCPETIAUX, Inspecteur Général des Prisons et des Etablissements de Bienfaisance, &c. . . . . 126

On the Mortality of Brussels, compared with that of other large Cities. By ED. DUCPETIAUX, &c.

ART. XIV.—1. Du Traitement de la Phthisie Pulmonaire. Quelques Reflexions sur les Phthisiques observés à l'Hôpital Saint-André de Bordeaux. Par EMILE L. PEREYRA, Médecin titulaire du dit Hôpital . . 140

On the Treatment of Pulmonary Consumption, &c. By EMILE L. PEREYRA, Physician to the Hospital St. André Bordeaux.

2. Observations on the Treatment of some forms of Incipient Phthisis. By JOSEPH BELL . . . . . . ib.

3. A Practical Inquiry into the value of Medicinal Naphtha in Tubercular Phthisis. By E. O. HOCKEN, M.D. . . . . . ib.

ART. XV.—Vestiges of the Natural History of Creation . 155

PAGE

ART. XVI.—De l'Emploi de la Chaleur dans le Traitment des Ulcères, des Plaies après les Amputations et les grandes Opérations chirurgicales, de l'Hysterie, des Maladies de la Peau, du Rhumatisme, &c. Par M. le Dr. JULES GUYOT . . . . . . . 182
On the Employment of Heat in the Treatment of Ulcers, Wounds after Amputation and other great Surgical Operations, of Hysteria, Diseases of the Skin, &c. Par M. le Dr. JULES GUYOT.

ART. XVII.—Udkast til Lov om Medicinalvæsenet i Norge, &c. . . 188

Prospectus of a Medical Bill for Norway. Presented by the Royal Commission.

ART. XVIII.—1. A Bill for the better Regulation of Medical Practice throughout the United Kingdom. Prepared and brought in by Sir James Graham. Ordered to be printed, 7th August 1844 . . . . 193

2. Charter of the Royal College of Surgeons of England, granted September 14th, 1834 . . . . . . . . ib.

Bye-laws and Ordinances of the Royal College of Surgeons of England . ib.

3. A Statement of the Society of Apothecaries, on the subject of their Administration of the Apothecaries' Act . . . . ib.

An Address of the Society of Apothecaries to the General Practitioners of England and Wales, on the Provisions of the Bill "For the better Regulation," &c. . . . . . . ib.

4. A Manifesto by the Medical and Surgical Association of the Borough of Marylebone . . . . . . ib.

5. An Exposition of the Laws which relate to the Medical Profession in England, &c. By JOHN DAVIES, M.D. &c. . . . . ib.

6. Remarks on Medical Reform; in two Letters addressed to Sir James Graham. By Sir JAMES CLARK, Bart. M.D. F.R.S. . . . . ib.

7. An Introductory Discourse on the Duties and Conduct of Medical Students, &c. By Sir B. C. BRODIE, Bart. . . . . ib.

8. A Letter addressed to the Secretary of the North of England Medical Association. By T. C. CARTER, Esq. . . . . ib.

9. A Speech at the Derby Meeting of the Provincial Medical Association, &c. By CHARLES HASTINGS, M.D. F.G.S. . . . . ib.

ART. XIX.—On the Remedial Influence of Oxygen or Vital Air, Nitrous Oxide, and other Gases, Electricity and Galvanism, in restoring the healthy functions of the principal Organs of the Body and the Nerves supplying the Respiratory, Digestive, and Muscular Systems. By J. EVANS RIADORE, M.D. F.L.S, &c. . . . . . . . 232

ART. XX.—The Principles and Practice of Obstetric Medicine and Surgery, in reference to the Process of Parturition. By F. H. RAMSBOTHAM, M.D., Fellow of the Royal College of Physicians, &c. . . . 235

ART. XXI.—Miscellaneous Contributions to Pathology and Therapeutics, being a series of Original and Practical Papers on Rickets, Hydrocephalus, Impotence, and Sterility, Pulmonary Apoplexy, and Hemoptysis. By J. R. SMYTH, M.D. . . . . . . 237

---

### PART SECOND.—Bibliographical Notices.

ART. I.—Researches and Observations on the Causes of Scrofulous Diseases. By J. G. LUGOL, Physician to the Hospital St. Louis, &c. Translated from the French, with an Introduction, and an Essay on the Treatment of the principal varieties of Scrofula. By W. H. RANKING, M.D. Cantab., Physician to the Suffolk General Hospital . . . . 239

ART. II.—An Apology for the Nerves; or their Influence and Importance in Health and Disease. By Sir GEORGE LEFEVRE, M.D. &c. . . 240

PAGE

ART. III.—1. An Essay on the Nature, Causes, and Treatment of Deafness; and on the Diseases and Obstructions of the Cavity of the Tympanum, and of the Eustachian Tube, as causes of Deafness; and a new method of treating them by Medicated Vapour. With a List of Cases. By D. CRONIN, Surgeon 242

Observations on the efficacy of Traction in the Cure of Consumption and Asthma. With authentic Cases. By D. CRONIN, Surgeon to the Free Dispensary for Asthma, Consumption, and Diseases of the Heart, 94, St. Martin's Lane . . . . . . ib.

3. Observations on the Preparation and Administration of Arnica Montana, or Leopard's Bane, and Scrophularia Nodosa, in the Cure of Nervous Deafness and Palpitation of the Heart. By D. CRONIN, M.D. &c. . . ib.

ART. IV.—Elements of Anatomy. By ALEX. LIZARS, M.D. &c. . . 243

ART. V.—The Anatomist's Vade-Mecum: a System of Human Anatomy. By ERASMUS WILSON . . . . . . ib.

ART. VI.—Treatise on Inflammation as a Process of Anormal Nutrition. By J. H. BENNETT, M.D. F.R.S.E. &c. . . . . 244

ART. VII.—Principles of Human Physiology, with their chief applications to Pathology, Hygiene, and Forensic Medicine. By W. B. CARPENTER, M.D. F.R.S. 245

ART. VIII.—A Dictionary of the Terms used in Medicine and the Collateral Sciences. By R. D. HOBLYN, A.M. Oxon. . . . . ib.

ART. IX.—A Manual of Elementary Chemistry, Theoretical and Practical. By GEORGE FOWNES, PH.D. &c. . . . . . ib.

ART. X.—1. The Medical Remembrancer, or Book of Emergencies. By E. B. L. SHAW, Surgeon . . . . . . 246

2. The Prescriber's Pharmacopœia. By a Practising Physician . . ib.

ART. XI.—Facts and Observations in Medicine and Surgery. By J. GRANTHAM 247

ART. XII.—Remarks on the use of Vivisection, as a means of Scientific Research. By RD. JAMESON. . . . . . . 248

---

### PART THIRD.—Original Reports and Memoirs.

1. Report on the Progress of Human Anatomy and Physiology, in the years 1843-4. By JAMES PAGET . . . . . . 249

2. Notices of the Lunatic Asylums of Paris. In a Letter to John Forbes, M.D. F.R.S. By JOHN CONOLLY, M.D., Fellow of the Royal College of Physicians, Physician to the County Lunatic Asylum at Hanwell . . . 281

3. On the Reflex Function of the Brain. By T. LAYCOCK, M.D. . . 298

---

Obituary Notice of the late Dr. Abercrombie . . : 312

" " Sir Henry Halford . . . 315

BOOKS RECEIVED FOR REVIEW . . . . . 316

